# Association of Molecular Classification with FIGO Stage and Survival Outcomes in Endometrial Cancer

**DOI:** 10.3390/medicina62050846

**Published:** 2026-04-29

**Authors:** Merve Keskinkılıç, Gül Polat, Zeynep Bayramoğlu, Anıl Aysal Ağalar, Göksenil Bülbül Öztürk, Emine Çağnur Ulukuş, Tuğba Yavuzşen, İlhan Öztop

**Affiliations:** 1 Department of Medical Oncology, Buca Seyfi Demirsoy Research and Training Hospital, İzmir Democracy University, Izmir 35390, Türkiye; mervekeskinkilic90@gmail.com; 2 Department of Medical Oncology, Faculty of Medicine, Dokuz Eylul University, Izmir 35330, Türkiye; polatgul19@gmail.com; 3 Department of Medical Pathology, Faculty of Medicine, Dokuz Eylul University, Izmir 35330, Türkiye; drzeynepbayramoglu@hotmail.com (Z.B.); anil.aysal@deu.edu.tr (A.A.A.); 4 Department of Medical Pathology, İzmir Atatürk Research and Training Hospital, Katip Çelebi University, Izmir 35360, Türkiye; goksenil.bulbul.ozturk@ikc.edu.tr; 5 Memorial Health Group, Department of Medical Pathology, Istanbul 34758, Türkiye; emine.ulukus@memorial.com.tr; 6 Department of Medical Oncology, Institute of Oncology, Dokuz Eylul University, Izmir 35330, Türkiye; drtugba@yahoo.com (T.Y.); ilhan.oztop@deu.edu.tr (İ.Ö.)

**Keywords:** dMMR, endometrium cancer, NSMP, molecular classification, POLEmut, p53abn

## Abstract

*Background and Objectives*: Molecular classification has emerged as a key determinant of prognosis in endometrial cancer and has recently been incorporated into the 2023 FIGO staging system. Tumors are categorized into four molecular subgroups—POLE-mutated (POLEmut), p53-abnormal (p53abn), mismatch repair-deficient (dMMR), and no specific molecular profile (NSMP)—each associated with distinct biological behavior and clinical outcomes. However, real-world data evaluating the relationship between molecular classification, FIGO stage distribution, and survival outcomes remain limited. *Materials and Methods*: This retrospective study included patients diagnosed with endometrial cancer between 2014 and 2022 at Dokuz Eylül University Hospital. Tumor samples were classified according to the ProMisE molecular algorithm using next-generation sequencing for POLE mutations and immunohistochemical evaluation of mismatch repair proteins and p53 expression. Clinicopathological characteristics, recurrence patterns, and survival outcomes were analyzed. Appropriate statistical analyses were performed. *Results:* A total of 156 patients were included (mean age 60.2 ± 10.0 years). The most common histology was endometrioid carcinoma (51.9%). Molecular subgroup distribution was NSMP (58.3%), dMMR (25%), p53abn (11.5%), and POLEmut (5.1%). Most patients presented with early-stage disease (83.4%). According to the 2023 FIGO molecular staging, 8.3% were classified as stage 2C m-p53abn and 5.8% as Stage 1Am-POLEmut. After a median follow-up of 39.5 months, the overall survival rate was 81.6%. Survival differed significantly across molecular subgroups, with the most favorable outcomes observed in the POLEmut (100%), followed by NSMP (85.2%), dMMR (78.4%), and p53abn (64.7%) (*p* = 0.011). Lymph node metastasis was significantly more frequent in the p53abn subgroup (*p* = 0.002), whereas distant metastasis rates did not differ between groups. *Conclusions:* Molecular classification was associated with differences in FIGO stage distribution and survival outcomes in this retrospective cohort and may provide additional prognostic information beyond traditional clinicopathological factors. The integration of molecular profiling into routine practice and staging systems may enable improved risk assessment and facilitate more personalized therapeutic strategies in endometrial cancer.

## 1. Key Messages

• What is already known?

Molecular classification of endometrial cancer has prognostic significance and was incorporated into the 2023 FIGO staging system, particularly for POLEmut and p53abn tumors.

• What does this study add?

In this single-center, real-world cohort, ProMisE molecular subgroups were associated with differential survival outcomes, with POLEmut tumors showing excellent prognosis and p53abn tumors demonstrating the poorest survival and higher lymph node involvement.

• How does this study affect research, practice, or policy?

These findings provide additional real-world evidence supporting the evolving role of molecular classification in risk stratification and personalized management strategies in endometrial cancer.

## 2. Introduction

Endometrial cancer is the most common gynecologic malignancy in developed countries, with both incidence and mortality continuing to rise globally [1]. Most patients are diagnosed at an early stage, and prognosis has traditionally been estimated using clinicopathologic parameters such as FIGO stage, histologic subtype, tumor grade, depth of myometrial invasion, and lymphovascular space invasion (LVSI) [2]. Although these factors remain central to treatment decision-making, they inadequately reflect the biological heterogeneity of endometrial cancer, leading to substantial variability in clinical outcomes among patients with similar clinicopathologic profiles [2].

A major paradigm shift in endometrial cancer risk stratification emerged with the molecular classification proposed by The Cancer Genome Atlas (TCGA), which identified four distinct molecular subgroups: POLE mutated (POLEmut), mismatch repair-deficient (dMMR), p53-abnormal (p53abn), and no specific molecular profile (NSMP) [3]. This molecular taxonomy has demonstrated strong and reproducible associations with prognosis, potentially improving prognostic discrimination beyond conventional histopathologic factors. POLEmut tumors are consistently associated with excellent outcomes, while p53abn tumors exhibit the poorest prognosis. dMMR and NSMP subgroups represent intermediate-risk categories, although their clinical behavior is heterogeneous [3,4].

To facilitate clinical implementation, the Proactive Molecular Risk Classifier for Endometrial Cancer (ProMisE) was developed, enabling molecular classification using a combination of POLE sequencing and immunohistochemical (IHC) assessment of mismatch repair proteins and p53 expression [4]. Subsequent studies, including analyses from the PORTEC-3 trial, confirmed that molecular classification provides independent prognostic information and predicts differential benefit from adjuvant therapies, particularly in high-risk disease [5]. These findings have led to the widespread integration of molecular classification into clinical trials and treatment algorithms [6].

Reflecting this growing body of evidence, molecular classification has been incorporated into international guidelines, including the ESGO/ESTRO/ESP recommendations [7], and into the revised FIGO 2023 staging system [8]. Recent contemporary reviews have further highlighted the paradigm shift toward molecularly integrated staging and its implications for routine clinical practice [9,10,11]. This updated framework recognizes molecular subtype as a critical determinant of prognosis and allows stage migration based on adverse molecular features, particularly p53 abnormalities. Recent studies have shown that the integration of molecular classification into staging systems improves prognostic discrimination compared with traditional FIGO staging alone, especially in early and intermediate stages of endometrial cancer [8,12]. More recent validation studies published after the implementation of the FIGO 2023 criteria have confirmed superior prognostic stratification and stage-specific survival discrimination with molecularly integrated staging systems [13,14].

Despite these advances, the prognostic relevance of molecular classification across different FIGO stages remains incompletely defined. Emerging evidence suggests that molecular subtypes interact with stage-specific clinicopathologic factors, such as lymph node involvement and depth of invasion, influencing recurrence patterns and survival outcomes [12,15]. In patients with lymph-node–positive disease, molecular classification has been shown to further stratify recurrence risk beyond nodal status alone [15]. Moreover, the molecular profile appears to predict patterns of recurrence, including locoregional versus distant failure, underscoring its potential role in tailoring adjuvant treatment strategies [16].

The prognostic impact of molecular classification is particularly relevant in tumors exhibiting multiple molecular features, a subgroup that represents a diagnostic and therapeutic challenge [17]. In addition, abnormal p53 expression has been identified as an adverse prognostic marker even in low-grade, early-stage endometrioid carcinomas, suggesting that molecular risk factors may override favorable clinicopathologic characteristics [18]. Conversely, patients with POLEmut tumors demonstrate excellent outcomes across stages, raising questions regarding potential overtreatment in this subgroup [19,20].

Taken together, these findings highlight the critical role of molecular classification in refining prognostic assessment in endometrial cancer. However, data evaluating its stage-specific prognostic value in real-world retrospective cohorts remain limited. The present study aims to evaluate the association of molecular classification with FIGO stage distribution and survival outcomes in patients with endometrial cancer, thereby providing further insight into the interaction between molecular subtype and disease stage and supporting more precise, biology-driven risk stratification.

## 3. Materials and Methods

### 3.1. Study Design and Population

In this retrospective study, patients diagnosed with endometrial cancer between 2014 and 2022 at the Dokuz Eylül University Faculty of Medicine, Department of Internal Medicine, Medical Oncology Department. Inclusion criteria for the study were as follows: (i) having a histologically confirmed diagnosis of endometrial cancer, (ii) being under follow-up for at least 3 months, (iii) having sufficient paraffin tissue belonging to the patient, (iv) having complete data, and (v) being female and 18 years of age or older. Exclusion criteria were as follows: (i) having a second malignancy, (ii) incomplete pathology reports, (iii) incomplete or inadequate IHC staining results, and (iv) discontinuation of follow-up immediately after surgery.

All patients meeting the study criteria were included consecutively. Therefore, since all patients meeting the inclusion criteria were included, patient selection or bias was not a factor. Demographic and clinicopathological characteristics at the time of diagnosis, CA 125 values, treatment, last follow-up, and death information were retrospectively recorded from the hospital database.

### 3.2. Ethical Approval

This study was designed retrospectively in accordance with the principles of the Helsinki Declaration, and participant data were used with the permission of the hospital administration. Informed consent was obtained from all subjects involved in the study. Based on this, the study was approved by the Non-Interventional Research Ethics Committee of Dokuz Eylül University Faculty of Medicine (Date: 22 May 2024/No: 2024/18-13). The reporting of this study complies with the STROBE Statement.

### 3.3. Tissue Samples and Molecular Classification Criteria

Molecular classification was performed according to the ProMisE algorithm in patients meeting the inclusion criteria. Formalin-fixed paraffin-embedded (FFPE) tumor sections from patients diagnosed with endometrial cancer were retrieved from the Medical Pathology Department archives for molecular analysis. DNA was extracted from deparaffinized tumor tissue using the Cobas^®^ High Pure FFPET DNA Isolation Kit (Roche Diagnostics International Ltd, Rotkreuz, Switzerland) according to the manufacturer’s instructions. DNA quantity and quality were assessed using Qubit fluorometric measurement to determine suitability for next-generation sequencing (NGS). Following library preparation and PCR amplification, sequencing was performed using the Illumina^®^ MiniSeq™ (San Diego, CA, USA) platform in accordance with standard Illumina protocols.

POLE mutation analysis targeted hotspot regions within the exonuclease domain of the POLE gene (exons 9–14). Pathogenic POLE variants were classified according to published pathogenicity criteria and available annotation databases, and only known pathogenic or likely pathogenic exonuclease domain mutations were considered POLE-mutated for molecular classification purposes. Tumors harboring pathogenic POLE mutations were assigned to the POLE ultramutated subgroup.

For tumors without pathogenic POLE mutations, immunohistochemical assessment of mismatch repair proteins (MLH1, PMS2, MSH2, and MSH6) was performed. Mismatch repair deficiency (dMMR) was defined as a complete loss of nuclear staining of one or more MMR proteins in tumor cells in the presence of intact internal positive control staining in stromal and inflammatory cells. Tumors demonstrating MMR deficiency were classified as the dMMR subgroup.

Tumors with intact MMR protein expression were subsequently evaluated for p53 expression by immunohistochemistry. p53 abnormality was defined according to validated interpretation criteria, including diffuse strong nuclear overexpression, complete absence of staining (null pattern), or unequivocal cytoplasmic staining. Tumors with aberrant p53 staining were classified as p53-abn, whereas tumors lacking these features were categorized as no specific molecular profile (NSMP).

In cases with multiple molecular classifier features, tumors were assigned according to the hierarchical ProMisE algorithm (POLEmut > dMMR > p53abn > NSMP). No multiple-classifier tumors were identified in the present cohort.

### 3.4. Statistical Analysis

Demographic characteristics, clinicopathological features, and blood sample results were collected from the hospital database. The ShapiroWilk test was used to evaluate the conformity of the data to a normal distribution, and the Levene test was used to evaluate the homogeneity of variance. In comparing two independent groups according to quantitative data, the independent-Sample *T* test was used with Bootstrap results, while the MannWhitney U test was used with the Monte Carlo simulation technique. In comparing categorical variables with each other, Pearson chi-square, linear-by-linear association, and Fisher–Freeman–Holton tests were employed using the Monte Carlo simulation technique and Fisher’s exact test results, and the column ratios were compared with each other and expressed according to the Benjamini–Hochberg corrected *p* value results. Overall survival was defined as the time from the date of diagnosis to death or the last follow-up. The median follow-up time in the study was calculated using the reverse Kaplan–Meier method. Kaplan–Meier (product limit method) and LogRank (MantelCox) analysis were used to examine the effects of variables on survival times. Potential prognostic risk factors were primarily evaluated using univariate Cox proportional hazards regression analyses. Although multivariable modeling was initially considered, the limited number of survival events in the cohort (*n* = 22) precluded the construction of a reliable, fully adjusted multivariable model without substantial risk of overfitting. Therefore, survival associations identified in regression analyses should be interpreted as exploratory and hypothesis-generating. Quantitative variables are expressed as mean ± standard deviation or median (minimum − maximum/interquartile range), while categorical variables are presented as *n* (%).

All statistical analyses were performed with the SPSS Statistics 24.0 for iOS software program (SPSS, Inc., Chicago, IL, USA), and variables were evaluated at a 95% confidence interval, and statistical significance was determined as *p* < 0.05.

## 4. Results

The mean age of the 156 patients included in the study was 60.2 ± 10.0 (range: 36–83). The most common histological subtype was endometrioid carcinoma, accounting for 51.9% (*n* = 81), and the vast majority of patients (83.1%, *n* = 113) had pathological grade 2. The vast majority of patients (83.4% (*n* = 130)) were in the early stage (49.4% (*n* = 77) Stage 1A, 14.1% (*n* = 22) Stage 1B, 19.9% (*n* = 31) Stage 2). Clinicopathological characteristics of the patient population are shown in Table 1.

When the treatment characteristics of the 156 cases included in the study were evaluated, it was determined that 28.2% (*n* = 44) of the patients received systemic treatment, 60.9% (*n* = 95) received radiotherapy, and 59.6% (*n* = 93) received brachytherapy, according to the stage of the disease at the time of diagnosis.

The most common subgroup in terms of molecular classification was NSMP with 58.3% (*n* = 91), followed by dMMR with 25% (*n* = 39), P53 abn with 11.6% (*n* = 18), and POLE mut with 5.1% (*n* = 8). According to the FIGO 2023 molecular classification, 8.3% (*n* = 13) of these patients were in Stage 2C m-p53abn, and 5.8% (*n* = 9) were in Stage 1Am-POLEmut.

When the distribution of molecular subgroups according to stages was evaluated, 87.5% of patients with POLEmuts, who had a good prognosis, were in Stage 1, while the vast majority of patients with p53abn, who had a poor prognosis (38.9%), were in Stage 3. The incidence of the p53 abnormality, which has a poor prognostic effect, increased statistically significantly as the stage progressed (*p* = 0.004). This is shown in Table 2.

When the relationship between regional and distant metastasis status and molecular subgroups was evaluated, lymph node involvement was highest in the p53abn group at 33.3%, and this difference was statistically significant (*p* = 0.002). Lymph node involvement was detected in 12.5%, 7.7%, and 3.4% of the POLEmut, dMMR, and NSMP groups, respectively.

No significant difference was found between molecular groups in terms of distant organ metastasis (*p* = 0.695).

The median follow-up period for the study was 39.5 months (11–62.8 months), and according to the final assessment at the data cutoff point, 14.1% (*n* = 22) of the patient population had died, and 85.9% (*n* = 134) were still alive.

**Figure 1:**
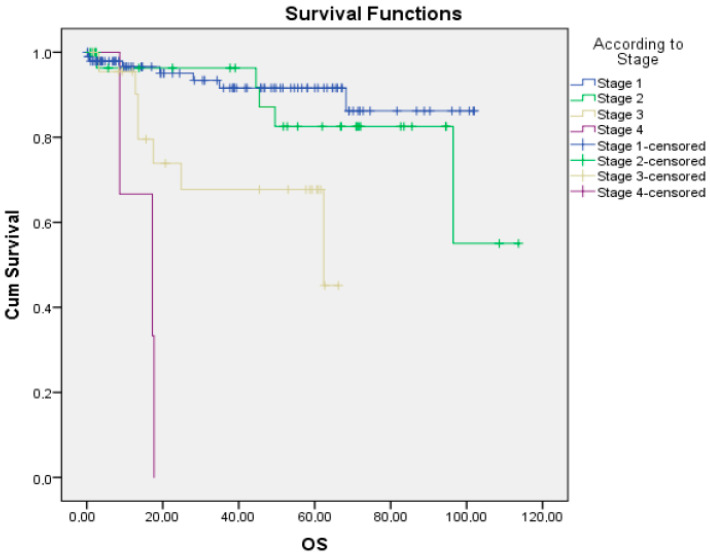
Overall survival according to stage (Kaplan–Meier test).

The overall survival time in the entire population was found to be a mean of 93.7 ± 3.8 months (95% CI 86.1–101.4 months). When the distribution of overall survival time according to stages was evaluated, it was found to be 93.1 ± 3.2 months in the Stage 1 group (95% CI 86.7–99.4 months), 95.5 ± 6.9 months in the Stage 2 group (95% CI 82.7–109.1 months), 48.7 ± 5.5 months in the Stage 3 group (95% CI 37.8–59.6 months), and 14.5 ± 3.8 months in the Stage 4 group (95% CI 8.7–20.3 months) (*p* = 0.0001) (Figure 1).

**Figure 2:**
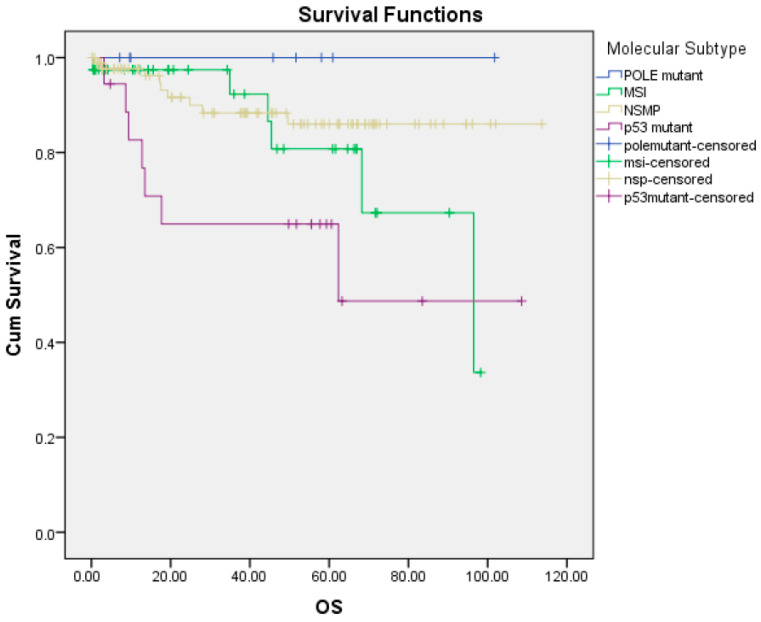
Overall survival according to molecular subtype (Kaplan–Meier test).

When overall survival rates were evaluated according to molecular subgroups, the group with POLEmut (100%) had the longest survival rate, followed by the NSMP group (90%), the dMMR group (84.6%), and the p53abn group (61.1%) (*p* = 0.025) (Figure 2).

The p53abn rate was statistically higher in the deceased patient group compared to the surviving patient group (40.9% vs. 12.7%, respectively; *p* = 0.003). In molecular subclassification, p53abn (31.8%) and dMMR (27.3%) rates were higher in the deceased patient group, while NSMP (61.2%) was dominant in the surviving patient group (*p* = 0.016). POLE mutation was not observed in the deceased patient group, whereas it was observed in 5.1% of the surviving patient group. There was no significant association between the presence of POLE mutation and survival (*p* = 0.594).

Exploratory analyses in the subgroup of patients receiving systemic treatment did not demonstrate statistically significant survival differences according to molecular subtype; however, given the limited sample size and small number of events in this subgroup, these findings should be interpreted with caution and are not suitable for definitive comparative inference.

Factors affecting overall survival were evaluated using univariate Cox regression analysis. The risk of death increased significantly with increasing age at diagnosis (HR = 1.092; 95% CI: 1.039–1.148; *p* = 0.001). Regarding histological subtypes, serous histology (HR = 3.36; *p* = 0.023), carcinosarcoma (HR = 25.30; *p* < 0.001), and clear cell histology (HR = 28.30; *p* = 0.003) were associated with a significant increase in risk, using endometrioid carcinoma as the reference. Pathological features such as endocervical involvement (HR = 2.53; *p* = 0.031), cervical involvement (HR = 2.43; *p* = 0.047), serosal involvement (HR = 3.91; *p* = 0.005), omental involvement (HR = 8.81; *p* < 0.001), ovarian involvement (HR = 3.70; *p* = 0.019), parametrium involvement (HR = 5.45; *p* = 0.007), presence of lymphovascular invasion (HR = 3.86; *p* = 0.004), and p16 positivity (HR = 7.14; *p* = 0.025) were significant prognostic factors negatively affecting survival. Additionally, lymph node metastasis (HR = 4.69; *p* = 0.003) and distant metastasis (HR = 15.89; *p* = 0.011) significantly reduced survival. Distant organ metastases, particularly lung (HR = 19.77; *p* = 0.006) and bone metastases (HR = 19.77; *p* = 0.006), dramatically increased the risk of death. Overall, the presence of metastases was also found to be a strong poor prognostic risk factor (HR = 6.96; 95% CI: 2.70–17.97; *p* < 0.001).

On the other hand, no significant association was found between myometrial involvement, abdominal washing fluid, ER/PR status, grade, systemic treatment, radiotherapy, and brachytherapy and survival (all *p* > 0.05).

In the FIGO staging, Stages 3A (HR = 4.86; *p* = 0.032), 3B (HR = 18.27; *p* = 0.009), 3C2 (HR = 7.80; *p* = 0.015), and 4A (HR = 30.87; *p* < 0.001) were highly unfavorable prognostic factors.

When molecular subgroups were evaluated using the POLEmut group as the reference, the risk of death was 1.7-fold higher in the NSMP group, although this difference was not statistically significant (HR = 1.655; 95% CI: 0.588–4.657; *p* = 0.340). No significant difference was observed in the dMMR group (*p* = 0.985). In contrast, the risk of death was approximately 3.3-fold higher in p53abn cases and was statistically significant (HR = 3.258; 95% CI: 1.211–8.760; *p* = 0.019). Among molecular markers, p53 abnormality was significantly associated with increased mortality risk in exploratory survival analyses (HR = 3.62; 95% CI: 1.54–8.49; *p* = 0.003). MMR status was not found to be significant (*p* > 0.05).

## 5. Discussion

In this retrospective cohort study, we evaluated the association of molecular classification with FIGO stage distribution and survival outcomes in patients with endometrial cancer and demonstrated that molecular subgroups were associated with clinically meaningful differences in stage distribution and survival outcomes beyond conventional staging parameters. Our findings are consistent with prior reports suggesting that p53-abnormal tumors are associated with a more advanced stage at diagnosis, increased lymph node involvement, and significantly inferior overall survival, whereas POLEmut tumors exhibit excellent outcomes regardless of stage. These observations are consistent with TCGA-based analyses and large validation cohorts showing that molecular subtype appears to be an important biological determinant of prognosis, often outweighing traditional clinicopathologic risk factors [3,4,5,8].

Consistent with previous real-world studies, NSMP constituted the largest molecular subgroup in our cohort, followed by dMMR, p53abn, and POLEmut tumors [4,6,12]. The predominance of NSMP tumors mirrors distributions reported in population-based and multicenter series and highlights the biological heterogeneity of this subgroup [6,12]. Although NSMP tumors are conventionally categorized as intermediate risk, our survival analyses demonstrated more favorable outcomes compared with p53abn tumors, supporting emerging evidence that prognosis within the NSMP group is strongly modulated by accompanying clinicopathologic features such as stage, lymphovascular invasion, and nodal status [12,16].

A key finding of our study is the strong association between p53 abnormality and advanced FIGO stage. The proportion of p53abn tumors increased significantly with stage progression, and p53abn cases were predominantly observed in Stage III–IV disease. This finding is in line with multiple studies demonstrating that p53 abnormality is linked to aggressive tumor biology, genomic instability, early dissemination, and poor survival outcomes [5,11,15,18]. Importantly, p53 abnormality was significantly more frequent among deceased patients and was associated with adverse survival outcomes in exploratory survival analyses, reinforcing its central role as a marker of high-risk disease across histologic subtypes and stages [11,18].

The association between molecular classification and lymph node involvement observed in our cohort further underscores the aggressive nature of p53abn tumors. Lymph node metastasis was significantly more common in the p53abn subgroup, consistent with multicenter retrospective studies showing that molecular classification refines prognostic stratification even among node-positive patients [15,21]. These findings are consistent with the hypothesis that molecular profiling may help identify patients with biologically aggressive disease who could be considered for more individualized adjuvant treatment and closer surveillance strategies [7,15].

In contrast, POLEmut tumors in our cohort were predominantly diagnosed at early stages and demonstrated excellent survival outcomes, with no deaths observed during follow-up. These results are concordant with multiple retrospective series and meta-analyses reporting near-perfect survival rates in POLEmut endometrial cancer, even in the presence of traditionally high-risk clinicopathologic features [19,20,22]. Our findings further contribute to the growing body of evidence suggesting that patients with POLEmut tumors may be overtreated when managed according to conventional risk stratification algorithms, supporting ongoing investigation of treatment de-escalation strategies in this subgroup [19,22].

Although molecular classification significantly stratified overall survival in the entire cohort, it did not demonstrate a discriminatory effect among patients receiving systemic therapy. This observation may be explained by the relatively small number of patients treated with systemic therapy, treatment heterogeneity, or the overriding prognostic influence of advanced stage and metastatic burden in this subset. Similar findings have been reported in real-world cohorts and post hoc analyses, emphasizing that while molecular classification is prognostic, its predictive value for treatment response remains less clearly defined and warrants further prospective investigation [5,6,23].

Stage-based survival analyses in our study confirmed the strong adverse prognostic impact of advanced disease, with markedly reduced survival in stage III and IV patients. However, the integration of molecular classification provided additional prognostic granularity by identifying biologically aggressive p53abn tumors within earlier stages and favorable POLEmut tumors across stages. These results align with recent studies demonstrating that molecularly integrated staging systems outperform traditional FIGO staging alone [8,12]. This paradigm shift has been formally adopted in the FIGO 2023 staging system, which incorporates molecular features—particularly p53 abnormality and POLE mutation—into stage assignment [8]. Subsequent validation studies have demonstrated that this reclassification results in clinically meaningful stage migration and improved prognostic alignment between assigned stage and observed outcomes [13,14].

The incorporation of molecular classification into the 2023 FIGO staging system has substantially altered risk stratification in endometrial cancer by enabling biologically driven stage migration, particularly through the upstaging of p53-abnormal tumors and downstaging of POLEmut tumors. This reclassification reflects accumulating evidence that molecular features may supersede conventional clinicopathologic factors in determining prognosis. Several validation studies have demonstrated that patients reclassified according to FIGO 2023 exhibit survival outcomes more consistent with their molecularly assigned stage rather than their traditional anatomic stage, supporting the clinical relevance of this stage migration. In particular, p53-abnormal tumors are frequently upstaged due to their aggressive biology and poor survival despite otherwise favorable histopathologic characteristics, whereas POLEmut tumors may be downstaged because of their exceptionally favorable outcomes even in the presence of high-risk features. Importantly, this molecularly integrated staging framework may directly influence adjuvant treatment recommendations by preventing undertreatment of biologically aggressive tumors and reducing overtreatment in favorable molecular subsets. Therefore, the incorporation of molecular features into FIGO 2023 may enhance prognostic stratification beyond simple stage reassignment, and it reflects the ongoing shift toward more biology-informed therapeutic decision-making in endometrial cancer [8,12,18,19,22].

The clinical relevance of these findings lies in their potential to inform individualized treatment strategies. Emerging contemporary literature suggests that molecularly integrated staging may directly influence adjuvant treatment selection, enabling escalation in biologically aggressive p53-abnormal disease and de-escalation in favorable POLEmut tumors [10,14,24]. Molecular classification not only refines prognostic assessment but also correlates with recurrence patterns and metastatic behavior [16,21]. In accordance with previous reports, we observed that molecular subtype was associated with patterns of disease spread, particularly lymphatic dissemination in p53abn tumors, reinforcing its role in guiding adjuvant treatment decisions and follow-up strategies [16,21].

## 6. Limitations

This study is limited by its retrospective design, single-center setting, and the relatively small number of POLEmut cases, which reduced the statistical power of subgroup analyses. Subgroup analyses among patients receiving systemic treatment were limited by small sample size and insufficient event numbers, precluding robust assessment of differential survival outcomes according to molecular subtype in treated patients. In addition, the limited number of survival events precluded the construction of a fully adjusted multivariable Cox regression model and restricted formal stage-stratified survival analyses, thereby limiting definitive conclusions regarding independent prognostic effects and stage-specific outcome differences. Accordingly, the observed associations between molecular subtype, stage distribution, and survival should be interpreted cautiously and considered hypothesis-generating. Nevertheless, the use of standardized molecular classification and the inclusion of a real-world patient cohort enhance the clinical relevance of our findings.

## 7. Conclusions

In conclusion, our findings demonstrate that molecular classification is significantly associated with FIGO stage distribution and survival outcomes in endometrial cancer. While p53 abnormality was associated with advanced stage presentation, increased lymph node involvement, and lower survival, tumors with POLE mutations were predominantly detected in early-stage disease and showed favorable outcomes. These findings provide supportive real-world evidence for the ongoing integration of molecular profiling into contemporary risk assessment and staging frameworks while highlighting the need for larger prospective studies to validate stage-classified prognostic effects and better define the interaction between molecular subtype and disease stage and its potential implications for treatment stratification.

## Figures and Tables

**Table 1:** Clinicopathological characteristics of the patient population.

Characteristic	*n* (%)
**Histological subtype**	
Endometrioid	81 (51.9%)
Endometrial carcinoma with squamous differentiation	25 (16.0%)
Mucinous endometrioid	26 (16.7%)
Serous	17 (10.9%)
Carcinosarcoma	3 (1.9%)
Clear cell	1 (0.6%)
Squamous differentiation + mucinous	3 (1.9%)
**FIGO stage at diagnosis**	
1A	77 (49.4%)
1B	22 (14.1%)
2	31 (19.9%)
3A	10 (6.4%)
3B	1 (0.6%)
3C1	8 (5.1%)
3C2	4 (2.6%)
4A	3 (1.9%)
**Metastasis State**	
Regional lymph node	16 (10.1%)
Distant metastasis	8 (5.1%)
Peritoneal involvement	6 (3.9%)
Lung metastasis	1 (0.6%)
Bone metastasis	1 (0.6%)
**Grade**	
Endometrioid Grade 1	8 (5.8%)
Endometrioid Grade 2	113 (83.1%)
Endometrioid Grade 3	15 (11.0%)
**Lymphovascular invasion (LVI)**	
Absent	109 (69.9%)
Present	46 (29.5%)
Unknown	1 (0.6%)
**Myometrium involvement**	
None	29 (18.6%)
>50%	76 (48.7%)
<50%	51 (32.7%)

**Table 2:** Molecular subgroup distribution relationship according to stage.

FIGO Stage	NSMP *n* (%)	dMMR *n* (%)	P53abn *n* (%)	POLEmut *n* (%)	Total *n* (%)	*p* Value
Stage 1	62 (62.6%)	25 (25.3%)	5 (5.1%)	7 (7.1%)	%100	**0.004**
Stage 2	19 (61.3%)	8 (25.8%)	4 (12.9%)	0	%100	
Stage 3	9 (39.1%)	6 (26.1%)	7 (30.4%)	1 (4.3%)	%100	
Stage 4	1 (33.3%)	0	2 (%66.7)		%100	

## Data Availability

The datasets used and/or analyzed in this study are available upon reasonable request from the corresponding author.
